# Graduates of Rush Medical College—Session of 1861–62

**Published:** 1862-02

**Authors:** J. C. Taggart

**Affiliations:** Beloit, Wis.


### GRADUATES OF RUSH MEDICAL COLLEGE,

### SESSION OF 1861-62.

| NAMES. | STATES. | THESES. |
|---|---|---|
| Charles E. Allen | Illinois | ——— |
| Albert A. Ames | Minnesota | Diarrhœa. |
| Steph. G. Armstrong | Indiana | Dyspepsia. |
| Aurelius T. Bartlett | Illinois | Effect of mental influ. in disease. |
| Leonard L. Bennett | Illinois | Diabetes. |
| Geo. W. Beggs | Illinois | Man considered as a whole. |
| James Brown | Illinois | Out-door exercise. |
| Elejah W. Boyles | Illinois | Hydrastis Canadensis. |
| Wm. L. Cuthbert | Illinois | The Liver. |
| J. Griffin Conley | Wisconsin | Inflammation of Brain. |
| Wm. D. Carter | Illinois | Iodine. |
| Samuel M. Dunn | Iowa | Erysipelas. |
| Thos. G. Drake | Indiana | Medical Student. |
| Jas. B. Farrington | Tennessee | Erysipelas. |
| A. Z. Huggins | Iowa | Pneumonia. |
| Jacob H. Houser | Indiana | Camp Dysentery. |
| Riley B. Hayden | Michichigan | Electricity. |
| Jacob M. Hagey | Illinois | Inflammation. |
| Clark E. Loomis | Illinois | Mind and its influ. on man. |
| J. Meek Lanning | Iowa | Bilious Remittent Fever. |
| Geo. J. Monroe | Illinois | Scarlatina. |
| Wm. Meacher | Wisconsin | Phlegmonous Erysipelas. |
| Wm. McKnight | Illinois | Typhoid Fever. |
| Fordyce D. Millard | Wisconsin | Food and Respiration and their relations to animal heat. |
| Elmer Nichols | Illinois | Treat. of Tubercular Phthisis. |
| Wm. Rush Patton | Illinois | Diagnosis. |
| H. W. Richardson | Illinois | Relation of collateral science to medicine. |
| Chas. M. Richmond | Indiana | Perspiration. |
| Wm. R. Russell | Wisconsin | Duties of an accoucheur in natural labor. |
| Robt. E. Stevenson | Illinois | Opium. |
| Sam. B. Ten Brock | Illinois | Urinary Calculi. |
| J. Allen Torry | Wisconsin | Placenta Prævia. |
| A. H. Whipple | Wisconsin | Dyspepsia. |
| D. Bishop Wren | Ohio | Fractures. |
| John A. Ward | Illinois | Menstruation. |
| Egbert H. Winston | Wisconsin | Cinchona. |

HONORARY.

J. C. Taggart, Beloit, Wis.

